# The potential pathways underlying the association of propyl-paraben exposure with aeroallergen sensitization and EASI score using metabolomics analysis

**DOI:** 10.1038/s41598-021-83288-9

**Published:** 2021-02-12

**Authors:** Yujin Lee, Eun Lee, Dong Keon Yon, Hye Mi Jee, Hey Sung Baek, Seung Won Lee, Joo-Youn Cho, Man Yong Han

**Affiliations:** 1 Department of Clinical Pharmacology and Therapeutics, Seoul National University College of Medicine and Hospital, 101 Daehak-ro, Jongno-gu, Seoul, 03080 Republic of Korea; 2 Department of Pediatrics, Chonnam National University Hospital, Chonnam National University Medical School, Gwangju, Republic of Korea; 3 Department of Pediatrics, Seoul National University Hospital, Seoul National University College of Medicine, Seoul, Republic of Korea; 4 Department of Pediatrics, CHA Bundang Medical Center, CHA University School of Medicine, 59 Yatap-ro, Bundang-gu, Seongnam, Gyonggi-do 13496 Republic of Korea; 5 Department of Pediatrics, Kangdong Sacred Heart Hospital, Hallym University College of Medicine, Seoul, Republic of Korea; 6 Department of Data Science, Sejong University College of Software Convergence, Seoul, Republic of Korea; 7 Department of Biomedical Sciences, Seoul National University College of Medicine, Seoul, Republic of Korea

**Keywords:** Environmental sciences, Biomarkers, Diseases, Medical research, Pathogenesis

## Abstract

Propyl-paraben exposure is associated with aeroallergen sensitization, but its association with atopic dermatitis (AD) is inconclusive. No studies have been conducted on the metabolomic pathways underlying these associations. We investigated the associations between propyl-paraben exposure and aeroallergen sensitization, AD, and Eczema Area and Severity Index (EASI) score and identified the underlying pathways using untargeted metabolomics analysis. We enrolled 455 children in a general population study. Skin prick tests were performed with the assessment of EASI score. Urinary propyl-, butyl-, ethyl-, and methyl-paraben levels were measured. Untargeted metabolomics analysis was performed on the first and fifth urine propyl-paraben quintile groups. The highest urine propyl-paraben quintile group was associated with aeroallergen sensitization, but not with AD. Glycine, threonine, serine, ornithine, isoleucine, arabinofuranose, d-lyxofuranose, citrate, and picolinic acid levels were higher, whereas palmitic acid and 2-palmitoylglycerol levels were lower in the highest quintile propyl-paraben group, than in the lowest quintile group. The propyl-paraben-induced metabolic perturbations were associated with serine and glycine metabolisms, branched-chain amino acid metabolism, and ammonia recycling. Propyl-paraben exposure was associated with aeroallergen sensitization and EASI score, partially via metabolomic changes related with oxidative stress, mTOR, peroxisome proliferator-activated receptors pathway, aryl hydrocarbon receptor signaling pathways, and tricarboxylic acid cycle.

## Introduction

Parabens are widely used as synthetic preservatives in cosmetics, pharmaceuticals, and foods due to their antimicrobial properties^1^. Exposure to paraben can cause diverse health problems, including impaired reproductive function, metabolism, thyroid signaling, and immune responses^1–3^. Parabens are detected most commonly as forms of methyl and propyl in 99.1% and 92.7% respectively, which is affected by personal preferences regarding the products used^4^. Paraben can be exposed through various delivery routes, including skin or mucosal absorption and ingestion. The health effects induced by paraben exposure depend on the delivery route and differ based on the type of paraben^1^.

Previous studies have identified a positive association between paraben exposure and allergen sensitization in cross-sectional studies^2,5^. However, studies regarding the associations between paraben exposure and atopic dermatitis (AD), and its related symptoms are scarce^5^. Particularly, even though propyl-paraben can be easily absorbed through the dermis and thereby can cause skin inflammation, studies on the associations between propyl-paraben exposure and AD are lacking^6^. AD and allergen sensitization are linked with skin barrier dysfunction and play an essential role as an initial step of the atopic march^7,8^.

Untargeted metabolomics analysis can serve as a useful and powerful method to uncover the altered pathways underlying the complex interactions between environmental chemical exposure and the development of allergic diseases, while also contributing to the identification of prognostic and therapeutic biomarkers^9,10^. Although a few studies have applied untargeted metabolomics to investigate the metabolic signature in allergic diseases^11^, no studies have applied untargeted metabolomics to investigate the effect of propyl-paraben exposure on aeroallergen sensitization and skin inflammation, including AD. In addition, the findings obtained from the metabolomics study in adults are not representative of children with or without allergic diseases^12^.

Therefore, in this study, we investigated the associations between urinary propyl-paraben concentrations and aeroallergen sensitization, AD, and Eczema Area and Severity Index (EASI) score, considering the effects of propyl-paraben exposure on the skin^13^. In addition, we have evaluated the possible altered pathways contributing to the association between propyl-paraben exposure and aeroallergen sensitization, AD, and EASI score using the untargeted metabolomics approach.

## Results

### Characteristics of the study population

The demographic characteristics of the study population are described in Table S1. Of the 455 children enrolled in this study, 54.7% were boys. Population demographics in the highest and lowest quintile propyl-paraben groups are presented in Table 1 and Fig. S1. The prevalence of aeroallergen sensitization, EASI score, and blood periostin levels were higher in children from the highest quintile group of urine propyl-paraben than in children from the lowest quintile group.

**Table 1:** Demographic and clinical characteristics of the participants in the first and fifth quintile groups of urinary propyl-paraben levels.

Variables	Children in the first quintile of urine propyl paraben (n = 91)	Children in the fifth quintile of urine propyl paraben (n = 91)	*P* value
Age, years ± SD	10.9 ± 0.8	11.0 ± 0.8	0.443
Gender, n (%)			0.455
Female	37 (40.7)	43 (47.3)	
Male	54 (59.3)	48 (52.7)	
BMI categories*, n (%)			0.745
Normal	75 (82.4)	78 (85.7)	
Overweight	10 (11.0)	7 (7.7)	
Obese	6 (6.6)	6 (6.6)	
Aeroallergen sensitization^†^, n (%)			**0.034**
No	45 (50.6)	31 (34.8)	
Yes	44 (49.4)	58 (65.2)	
Atopic dermatitis^‡^, n (%)			0.430
No	78 (85.7)	73 (80.2)	
Yes	13 (14.3)	18 (19.8)	
EASI score, n (%)			0.061
Negative	82 (90.1)	73 (80.2)	
Positive	9 (9.9)	18 (19.8)	
Chitinase 3-like protein 1, ng/mL, median [IQR]	21.2 [16.6–28.2]	20.0 [16.0–28.6]	0.694
Periostin level, ng/mL, median [IQR]	31.4 [22.5–39.0]	38.4 [28.2–43.0]	**0.008**

*BMI* body mass index, *EASI* Eczema Area and Severity Index, *IQR* Interquartile range, *SD* standard deviation.

*BMI categories were classified using the BMI z-score. Obesity was defined as a BMI z-score of ≥ 1.62 and overweight was defined as 1.03 ≤ BMI z-score ≤ 1.61.

^‡^AD was defined as the presence of AD symptoms in the preceding 12 months.

^†^Missing = 4.

Data in bold means statistically significant values (*P* < 0.05).

### Associations between aeroallergen sensitization, AD symptoms in the previous 12 months, and urine propyl-paraben levels

Among the various paraben biomarker levels, the associations between aeroallergen sensitization and urine paraben concentrations were consistently significant only for propyl-paraben (Table 2; Table S2). When the lowest quintile group was considered as a reference group, the highest quintile of urine propyl-paraben levels was associated with aeroallergen sensitization (adjusted odds ratio [aOR], 2.021; 95% CI 1.093–3.738, *P* = 0.025; Table 2). Regarding AD symptoms in the previous 12 months, we found no significant association with the highest quintile of urinary propyl-paraben levels.

**Table 2:** Odds ratios (95% confidence intervals) for the associations between aeroallergen sensitization and atopic dermatitis symptoms in the previous 12 months by quintile levels of urinary propyl-paraben.

Urinary propyl-paraben level	OR (95% CI)	*P* value	aOR* (95% CI)	*P* value
**Aeroallergen sensitization**				
1st Quintile	Ref		Ref	
2nd Quintile	1.142 (0.636–2.049)	0.657	1.248 (0.685–2.276)	0.469
3rd Quintile	**1.979 (1.086**–**3.609)**	**0.026**	**2.083 (1.127**–**3.849)**	**0.019**
4th Quintile	**1.979 (1.086**–**3.609)**	**0.026**	**1.902 (1.028**–**3.517)**	**0.040**
5th Quintile	**1.913 (1.048**–**3.495)**	**0.035**	**2.021 (1.093**–**3.738)**	**0.025**
**Atopic dermatitis symptoms in the previous 12 months**				
1st Quintile	Ref		Ref	
2nd Quintile	1.442 (0.656–3.171)	0.362	1.442 (0.656–3.171)	0.362
3rd Quintile	1.011 (0.439–2.329)	0.980	1.011 (0.439–2.329)	0.980
4th Quintile	0.800 (0.336–1.907)	0.615	0.800 (0.336–1.907)	0.615
5th Quintile	1.479 (0.673–3.249)	0.330	1.479 (0.673–3.249)	0.330

*aOR* adjusted odd ratio, *CI* confidence interval, *OR* odds ratio, *Ref.* reference group.

Numbers in bold indicate a significant difference (*P* < .05).

*Logistic regression analysis was performed with adjustment for confounding factors, including age, gender, body mass index z-score, the presence of visible mold at home, and exposure to environmental tobacco smoke.

### Associations between EASI score and urinary propyl-paraben levels

To identify the associations between the severity and extent of skin inflammation in AD and urinary propyl-paraben levels, we measured EASI score of the study participants, regardless of the presence of AD. When the lowest quintile of urinary propyl-paraben levels was considered as a reference, the EASI score was significantly associated with the highest quintile group of urinary propyl-paraben, both in children with AD (aOR, 4.568; 95% CI 1.215–17.172, *P* = 0.025) as well as in all study participants (aOR, 2.441; 95% CI 1.014–5.876, *P* = 0.046; Table 3). However, there were no significant associations between EASI score and urinary levels of other types of paraben in the total population (Table S2).

**Table 3:** Association between urinary propyl-paraben levels and EASI score in the total population as well as children with atopic dermatitis.

Urinary propyl paraben levels	EASI score							
	All children				Children with atopic dermatitis symptoms in the previous 12 months			
	Crude OR (95% CI)	*P* value	aOR* (95% CI)	*P* value	Crude OR (95% CI)	*P* value	aOR* (95% CI)	*P* value
1st Quintile	*Ref*	–	*Ref*	–	*Ref*	–	*Ref*	–
2nd Quintile	1.701 (0.695–4.158)	0.244	1.802 (0.726–0.473)	0.204	1.684 (0.467–6.068)	0.425	1.909 (0.506–7.196)	0.340
3rd Quintile	1.970 (0.821–4.726)	0.129	1.863 (0.765–4.536)	0.171	3.000 (0.812–11.081)	0.099	3.112 (0.78812.290)	0.105
4th Quintile	1.402 (0.560–3.511)	0.471	1.441 (0.570–3.640)	0.440	1.429 (0.347–5.882)	0.621	1.895 (0.428–8.392)	0.400
5th Quintile	2.247 (0.951–5.309)	0.065	**2.441 (1.014**–**5.876)**	**0.046**	**3.500 (1.039**–**11.789)**	**0.043**	**4.568 (1.215**–**17.172)**	**0.025**

*aOR* adjusted odds ratio, *CI* confidence interval, *EASI* Eczema Ares and Severity Index, *Ref.* reference population group.

*P values calculated by logistic regression analysis for positive EASI score.

Numbers in bold indicate a significant difference (*P* < .05).

*Logistic regression analysis was performed with an adjustment for confounding factors, including age, gender, body mass index z-score, the presence of visible mold at home, and exposure to environmental tobacco smoke.

### Urinary metabolomics analysis with global metabolomic profiling

To identify the possible biomarkers and altered pathways linking propyl-paraben exposure with aeroallergen sensitization and EASI score, urinary metabolites in the highest and lowest quintiles of urinary propyl-paraben were analyzed and compared. We validated the reliability of the analytical performance by using the QC samples tightly clustered in the principal component analysis (PCA) plot (Fig. S2A). The global metabolomic profiling, which represents the differences in the distribution of the metabolomic datasets between the two groups, identified no apparent discrimination between the two groups.

**Figure 1:**
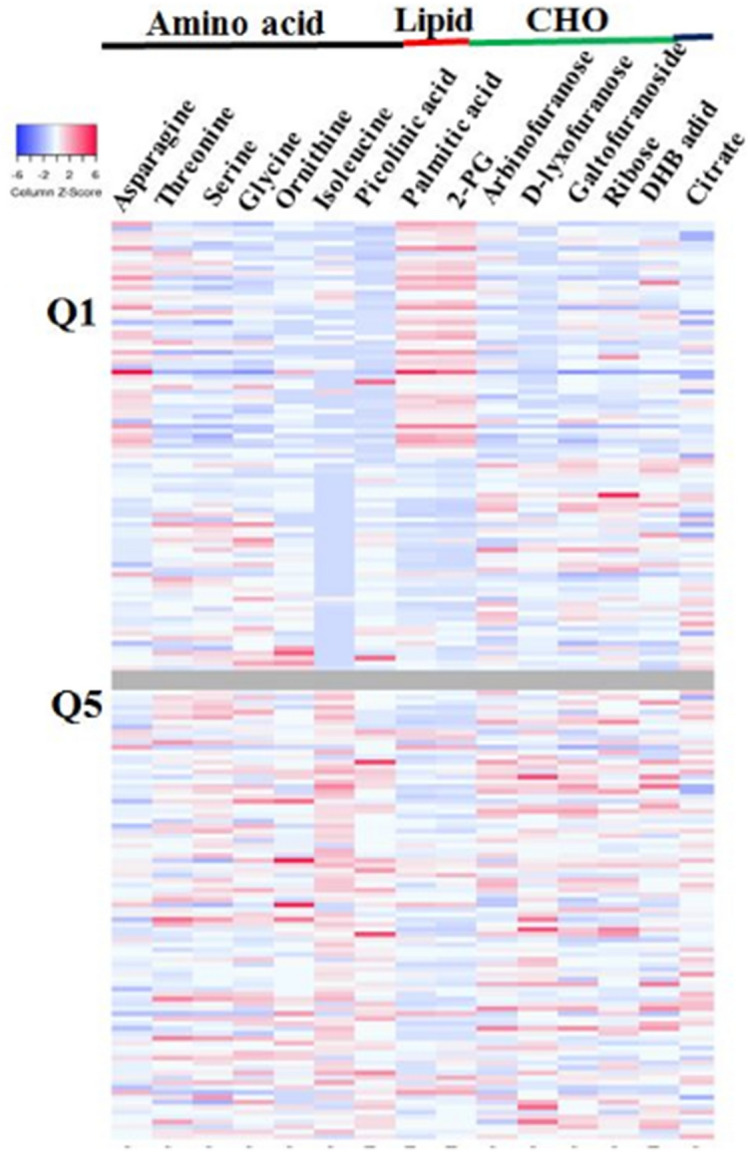
Heat map of urinary metabolites which significantly differ (*P* < .05) between children in the highest quintile (Q5) and those in the lowest quintile (Q1) groups of urinary propyl-paraben. Significantly different metabolites between the two groups are presented according to the amino acid, lipid, and carbohydrate metabolites. CHO, carbohydrate; Q1, lowest quintile group of urinary propyl-paraben; Q5, highest quintile group of urinary propyl-paraben.

We detected the 1543 metabolic features using gas chromatography time-of-flight mass spectrometry (GC-TOFMS). The features were involved in data processing; peak deconvolution, noise reduction, removal of artificial, and data normalization. Afterward, we performed statistical analysis using the processed data. As a result, six amino acids, two fatty acids, four carbohydrates, one citric acid, one glycolic acid, and one carboxylic acid were found to differ significantly between the two groups and they were identified through the identification of metabolic markers method (Figs. 1 and S4; Table S3).

### Possible pathways involved based on the metabolomics analysis

Among the 15 metabolites, glycine, threonine, serine, ornithine, isoleucine, arabinofuranose, d-lyxofuranose, ribose, ethyl d-galactofuranoside, citrate, and picolinic acid levels were higher in the highest quintile group of urinary propyl-paraben than in the lowest quintile group (Fig. 1, Fig. S2B; Table S3). Meanwhile, the levels and mean peak areas of asparagine, palmitic acid, and 2-palmitoylglycerol were lower in the highest quintile group of urinary propyl-paraben, than in the lowest quintile group. To determine the biological relevance of the associations between propyl-paraben exposure levels and metabolic changes, pathway enrichment analysis was performed. The 15 metabolites that changed significantly were involved in multiple perturbed metabolic pathways, including the metabolism of amino acids, carbohydrates, lipids, and tryptophan, as well as energy with the urea cycle (Fig. S2). In addition, the propyl-paraben induced metabolic perturbations were significantly related to glycine and serine metabolism and ammonia recycling (Figs. S3 and S4). These results indicate that exposure to higher propyl-paraben levels may lead to more defects in the glycine and serine metabolism and ammonia recycling than that associated with exposure to lower levels of propyl-paraben.

## Discussion

### Summary of the results of the present study

The present study found a significant association between aeroallergen sensitization, EASI score, and urinary propyl-paraben levels. However, we did not observe a significant association between AD and urinary propyl-paraben levels. Moreover, we identified the possible pathways that might underlie these associations using untargeted metabolomics analysis. However, there were no significant associations between levels of other types of parabens, including methyl-paraben, ethyl-paraben, and butyl-paraben, and aeroallergen sensitization, AD, and EASI score. The metabolites that differed between the highest and lowest quintile groups of urinary propyl-paraben included palmitic acid, 2-palmitoylglycerol, picolinic acid, and various hydroxyl acids, carbohydrates, and amino acids, suggesting that propyl-paraben exposure might disturb these pathways. The results of the present study might contribute to a better understanding of the associations among propyl-paraben exposure, aeroallergen sensitization, and EASI scores, while also providing suggestions, using an untargeted metabolomics approach, regarding the pathways that might underlie these associations. To our knowledge, this study is the first to identify the possible pathways that link aeroallergen sensitization, the degree of AD symptoms, and exposure to environmental parabens by using the untargeted metabolomics approach.

### The meaning of the results of the present study with comparison with previous studies

Thus far, there have been no studies demonstrating a positive association between paraben exposure and AD, although only one study showed that there were no significant associations between eczema and triclosan and parabens^5^. In our cross-sectional study, we did not observe a significant association between the presence of AD symptoms during the preceding 12 months and urinary propyl-paraben, methyl-paraben, ethyl-paraben, and butyl-paraben levels. However, EASI scores, which include the degree of erythema, edema or papulation, excoriation, and lichenification, as well as the extent of the skin affected, were associated with the highest quintile group of urinary propyl-paraben, when the lowest quintile group was used as a reference. The lack of consistency regarding the associations between AD, EASI score, and urinary propyl-paraben levels might be partially owing to the questionnaire-based investigation on the presence of AD symptoms in the preceding 12 months. In children with mild AD symptoms, AD symptoms might not be recognized without visiting a clinic. Moreover, the significantly increased levels of blood periostin in children in the highest quintile of urinary propyl-paraben levels when compared to those in the lowest quintile might support a positive association between the severity of AD symptoms and exposure levels of propyl-paraben, as children with severe and persistent AD have higher levels of blood periostin than those with mild AD^14,15^.

Since the EASI score was measured by pediatric allergists in all participants after confirmation of no intra- and inter-differences in scoring, the EASI score has its own significance, regardless of the self-reported AD symptoms. Skin barrier defects, indirectly reflected in the positive EASI score, might be linked with aeroallergen sensitization^16^. Therefore, the consistent association between aeroallergen sensitization, EASI score, and urinary propyl-paraben levels suggest that propyl-paraben exposure affects allergic outcomes, including aeroallergen sensitization and skin inflammation in AD. In addition, since assessment items in the EASI score include clinical signs of skin barrier dysfunction and might, therefore, predict the later development of AD as one of the steps in allergic march^17^, long-term follow-up studies are needed to determine whether the association between propyl-paraben exposure and allergic outcomes affects the prognosis of AD or leads to the development of other allergic diseases.

### Possible pathways underlying the associations between propyl-paraben exposure levels and allergic outcomes

**Figure 2:**
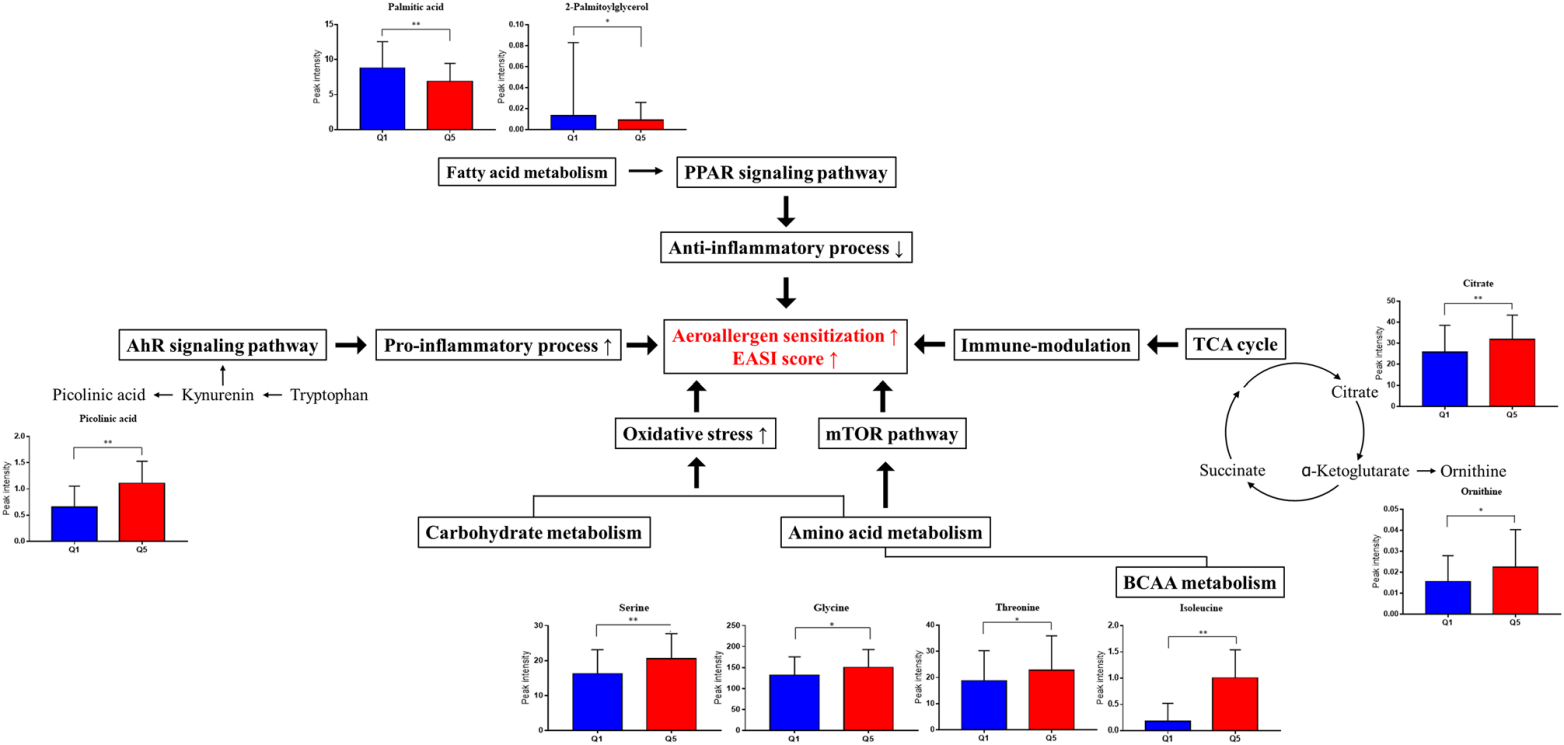
Possible pathways underlying the associations between urinary propyl-paraben levels and allergic outcomes. Our data suggest that at least four altered metabolic pathways might be correlated with propyl-paraben exposure and allergic outcomes. Q1, the lowest quintile of propyl-paraben levels; Q5, the highest quintile of propyl-paraben levels. *P < .05, **P < .001.

Our data suggest that at least five altered metabolic pathways might be linked to exposure to propyl-paraben and aeroallergen sensitization and EASI score (Figs. 2 and S5). First, changes in carbohydrates and amino acids levels, including asparagine, threonine, serine, glycine, and ornithine, cause oxidative stress^18^, thereby playing an essential role in the pathogenesis of allergic inflammatory skin disease and allergen sensitization^19–21^. In addition, paraben itself can generate reactive oxygen species and induce oxidative DNA damage^22^. Second, the mammalian target of rapamycin (mTOR) might be involved in the link between exposure to propyl-paraben and aeroallergen sensitization and EASI score. Amino acid levels, including glycine, serine, and isoleucine, were increased in the highest quintile group of urinary propyl-paraben than those in the lowest quintile group. Specifically, isoleucine levels, which is one of the branched-chain amino acids (BCAA), showed the most significant difference between the two groups (Table S3 and Fig. S4). Changes in the metabolites involved in the BCAA pathway are known to influence the regulation of mTOR^23,24^, which plays a vital role in epidermal barrier formation and the signaling axis for the control of filaggrin as the fundamental pathophysiology of AD^25–28^. Third, propyl-paraben exposure is associated with aeroallergen sensitization and increased EASI score via the peroxisome proliferator-activated receptors (PPAR) pathway, which is based on decreased levels of palmitic acid and 2-palmitoylglycerol found in our study^29,30^. The decreased levels of palmitic acid and 2-palmitoylglycerol in the highest quintile group of urinary propyl-paraben, compared with those in the lowest quintile group, can result in the reduced induction of the PPAR pathway, which might be linked with decreased anti-inflammatory responses, increased allergic skin inflammation in AD, and aeroallergen sensitization^29^.

Furthermore, the increased levels of picolinic acid, a catabolite of tryptophan metabolism and an interim metabolite of kynurenine, in the highest quintile group of urinary propyl-paraben might suggest that the aryl hydrocarbon receptor (AhR) signaling pathway might be involved in the pathophysiology of aeroallergen sensitization and increased EASI score in exposure to propyl-paraben. AhR is an active ligand transcription factor affected by metabolisms or pollutants^31^, and activation of this pathway plays a role in immune-modulation due to its action on diverse types of immune cells^31^. After the binding of AhR with propyl-paraben, the subsequent cascade of immunomodulation and epidermal differentiation might play a role in aeroallergen sensitization and increased EASI score^32–34^. Finally, the tricarboxylic acid (TCA) cycle, reflected in the altered citrate level found in this study, can shape immune cell responses via changes in the metabolic pathways of immune cells^35^, which may play a role in aeroallergen sensitization and increased EASI score.

### Limitations of the present study

Our study has several noteworthy limitations. First, the presence of AD symptoms in the preceding 12 months was assessed using the ISAAC questionnaire. However, the ISAAC questionnaire has been widely used in the epidemiologic studies of allergic diseases to identify the prevalence of allergic diseases, including AD^36^. Second, we did not investigate the direct mechanisms of action of propyl-paraben exposure on aeroallergen sensitization and increased EASI score. Instead, the pathways that possibly underlie the associations were inferred based on the untargeted metabolomics analysis results, which can measure as many urine metabolites as possible without bias^37,38^. Third, we could not conclude whether propyl-paraben exposure might induce the metabolites changes in a dose-dependent manner because only some key metabolites, including glycine, threonine, and palmitic acid, showed significant associations with propyl-paraben levels (data not shown). Fourth, the urinary metabolomics analysis was performed only in the lowest and highest quintile groups of urinary propyl-paraben. Thus, by investigating the changes in metabolites between the lowest and highest quintile, the differences in metabolites between groups with different propyl-paraben exposure levels can be maximized. Exposure to different types of paraben is affected by personal preference in terms of products^4^, and therefore, the associations can differ based on race and ethnicity. Moreover, further studies are needed to confirm our study results due to the small sample size.

### Conclusions

In conclusion, we have identified that exposure to propyl-paraben was associated with aeroallergen sensitization and increased EASI score, partially through the metabolomics changes linked with altered oxidative stress, PPAR pathway, AhR signaling pathway, and TCA cycle. These novel findings provide new insights into the relationship between propyl-paraben exposure, aeroallergen sensitization, and EASI score and might help develop therapeutic targets for inflammatory skin diseases caused by environmental pollutants.

## Methods

### Study population

This study was performed as a part of the general population-based study (Seongnam Atopy Project for Children's Happiness 2017), which were performed in 11 elementary schools in Seongnam City, South Korea^39–42^. Among the 620 children (10 to 12 years old), 455 (73.4%) children, which had complete information on the questionnaires, blood and urine samples and results of skin prick tests (SPTs) were enrolled in this study. The study protocol was approved by the Institutional Review Board of CHA University (2017-04-049) and written informed consent was obtained from all parents or guardians of the children who participated. All methods performed out in accordance with relevant guidelines and regulations.

### Questionnaire

The International Study of Asthma and Allergies in Childhood (ISAAC) questionnaire was used to investigate the presence of allergic diseases, including AD^43^. AD was defined as the presence of AD symptoms in the 12 months preceding the questionnaire and was investigated using the following question: “During the previous 12 months, have you had AD symptoms, such as itchy eczema?”.

### SPTs

SPTs were performed for eight common inhalant allergens, including *Dermatophagoides pteronyssinus*_,_ *Dermatophagoides farinae*_,_ *Alternaria*, cat epithelium, dog dander, birch, orchard grass, and Japanese hop. Aeroallergen sensitization was defined as at least one positive response (wheal diameter ≥ 3 mm) on SPTs^43^.

### EASI score

The EASI score is one of the most effective and reliable tools for the evaluation of AD^44,45^. To identify the skin barrier status, the EASI score was measured in all participants regardless of AD diagnosis ever or the presence of AD symptoms in the previous 12 months by pediatric allergists, who have no information regarding the presence of AD symptoms in the previous 12 months. The EASI score for each participant was measured in a closed space at the time of enrollment. Before measuring the EASI score, no significant difference was identified in the inter-rater reliability of the EASI score (*P* > 0.05). The EASI score was defined as negative or positive when the values were 0 or ≥ 0.1, respectively^46^.

### Assessment of urinary parabens concentrations

To avoid the possible confounding factors on the urinary paraben concentrations, urine samples were collected between 9 and 11 AM for all participants along with the measurement of the EASI score. Urinary concentrations of four parabens, including butyl-paraben, ethyl-paraben, methyl-paraben, and propyl-paraben, as well as creatinine concentrations, were measured from spot urine samples in all participants. The quantification of urinary parabens was performed according to the previously reported method, which used an Ultimate 3000 UHPLC system (Dionex, Sunnyvale, CA, USA) with a Thermo Scientific TSQ Quantiva Triple Quadrupole mass spectrometer (Thermo Scientific, San Jose, CA, USA) in multiple reaction monitoring modes^47^. The quantification limit for all analyzed parabens was 0.1 μg/L. The levels of each paraben were corrected using the urine creatinine concentrations to adjust for urinary dilution^48^.

### Measurement of blood periostin and chitinase levels

Serum periostin concentrations were measured using a proprietary sandwich enzyme-linked immunosorbent assay (Human Periostin/OSF-2 DuoSet ELISA; Shino-test, Kanagawa, Japan), which utilized anti-periostin antibodies (clones SS18A and SS17B)^41^. Serum YKL-40 levels were measured in duplicate using a commercial Human Chitinase 3-like 1Quantikine ELISA Kit according to the manufacturer’s instructions (R & D Systems, Inc. Minneapolis, MN, USA).

### Subjects for untargeted metabolomics analysis

The untargeted metabolomics analysis was performed in children from the lowest quintile (n = 91) and the highest quintile groups (n = 91) of urine propyl-paraben concentrations to identify the metabolic changes according to propyl-paraben exposure levels and to investigate the pathways underlying the associations between urine propyl-paraben levels, allergen sensitization, and EASI score.

### Urine sample preparation and gas chromatography time-of-flight mass spectrometry (GC-TOFMS) analysis

All urine samples were prepared using minor modifications of a protocol from the previous study^49^. The samples were thawed on ice and the quality control (QC) samples, used to validate the stability of the analytical performance, were prepared by pooling equal amounts of all urine samples. A 50 μL sample was extracted using 1 mL of the first extraction solution (3:3:2, acetonitrile:isopropanol:H_2_O). The samples were centrifuged for 10 min at 18,945 RCF, 4 °C, and 400 μL of the supernatant was dried under nitrogen. The samples were extracted with 400 μL of the second extraction solution (1:1, acetonitrile:H_2_O). Afterward, the extracted samples were dried under nitrogen. The dried samples were derivatized with methoxyamine (20 mg/mL in pyridine) at 30 °C for 90 min and subsequently trimethylsilylated with a mixture of fatty acid methyl ester (used for the retention time index) and N-methyl-N-(trimethylsilyl)-trifluoroacetamide (MSTFA) at 70 °C for 45 min. Lastly, prepared samples were analyzed using an Agilent 7890 series gas chromatography system (Agilent, Santa Clara, CA) coupled to a time-of-flight mass spectrometer (LecoCorp., St. Joseph, MI, USA). An Rtx-5MS fused silica capillary column (30 m × 0.25 mm inner diameter and 0.25 μm film thickness) was used. A sample volume of 1 μL was split-injected for each analysis. Helium was used as the carrier gas, with a constant flow rate of 1.5 mL/min through the column. The GC oven temperature was maintained at 50 °C for 0.5 min, then increased to 330 °C at a rate of 20 °C/min and held for 5 min. The transfer line temperature was kept at 280 °C. Electron impact ionization (70 eV) at full scan mode (mass‐to‐charge ratio [m/z] 50–800) was used, with the ion source temperature at 250 °C.

### Metabolomics data analysis

Raw data were aligned using ChromaTOF (Leco, St. Joseph, MI) and peak areas were exported and processed using Metaboanalyst 4.0 open-source software (http://www.metaboanalyst.ca/)^50,51^. Artifacts and missing values were removed in > 20% of the samples and peak areas were normalized with the sum of metabolites for each sample. The processed data were entered into Metaboanalyst 4.0 for multivariate statistical analysis, including PCA and GraphPad Prism 7 (https://www.graphpad.com/scientific-software/prism) (GraphPad Software, Inc., San Diego, CA, USA) were used for univariate statistical analysis, including F-test, Student’s t-test, and measurement of fold change. *P *values of less than 0.05 were considered statistically significant.

### Identification of metabolic markers

The online HMDB database (https://hmdb.ca/)^52^, KEGG database (https://www.genome.jp/kegg/)^53^, and three commercially available libraries were used to identify metabolites; NIST, LECO-Fiehn Rtx5, and Wiley 9. The mass spectra of the markers were matched with the libraries and then, authentic standards were analyzed to compare the spectra with the markers. Finally, the retention time of standards was compared with the markers by calculating the relative retention index^49^.

### Statistical analysis

Data were analyzed using covariance or logistic regression analysis and presented as mean differences or odds ratios with 95% confidence intervals (CIs). The logistic regression analyses were performed after categorizing of the EASI score into two groups according to an EASI score of 0 or ≥ 0.1 to identify the associations between urine propyl-paraben levels and EASI score. The logistic regression analysis was performed to determine the association of quintile levels of urinary propyl paraben with aeroallergen sensitization and EASI score with adjustment for confounding factors, including age, gender, body mass index z-score, the presence of visible mold at home, and exposure to environmental tobacco smoke. Multivariate analysis and pathway analysis were performed to determine significantly different metabolites between the highest and lowest quintile urine propyl-paraben groups. Random forest was used for the analysis of the metabolome profiles. All data analyses were conducted using SPSS version 25.0 (IBM Co, Armonk, NY, USA). A *P*-value below 0.05 was considered statistically significant.

## Supplementary Information

Supplementary Information.

## Data Availability

The metabolomics data are available in the electronic Supplementary Material and at the NIH Common Fund’s National Metabolomics Data Repository (NMDR) website [Project ID: PR000925].
