# Supplementary material for: The potential pathways underlying the association of propyl-paraben exposure with aeroallergen sensitization and EASI score using metabolomics analysis
Source: Sci Rep. 2021 Feb 12;11:3772. doi: 10.1038/s41598-021-83288-9 (PMC7881090; doi:10.1038/s41598-021-83288-9)

**Supplementary Information**

**The potential pathways underlying the association of propyl-paraben exposure with aeroallergen sensitization and EASI score using metabolomics analysis**

Yujin Lee^1†^, Eun Lee^2†^, Dong Keon Yon MD^3^, Hye Mi Jee^4^, Hey Sung Baek^5^, Seung Won Lee^6^, Joo-Youn Cho^1,7*^ and Man Yong Han^4*^

The PDF file contains:

- Supplementary Figure 1-3

- Supplementary Table 1-4

**Supplementary Table 1.** Demographic and clinical characteristics of all participants

| Variables | Value |
| --- | --- |
| Number | 455 |
| Age, years (95% CI) | 11.0 (10.9–11.1) |
| Gender, males, n (%) | 249 (54.7) |
| BMI categories^*^, n (%) |  |
| Normal | 381 (83.7) |
| Overweight | 44 (9.7) |
| Obese | 30 (6.6) |
| Aeroallergen sensitization^†^, n (%) | 270 (59.9) |
| Atopic dermatitis^‡^, n (%) | 125 (27.7) |
| EASI score^§^ |  |
| Negative | 382 (84.7) |
| Positive | 69 (15.3) |

BMI, body mass index; CI, confidence interval; EASI, Eczema Area and Severity Index.

^*^BMI categories were classified using the BMI z-score.

^†^Aeroallergen sensitization means more than 1 positive response among the 8 allergens on skin prick tests. missing=4.

^‡^AD was defined as the presence of AD symptoms in the preceding 12 months.

^§^Missing = 4

**Supplementary Table 2.** Association of urinary quintile levels of each paraben with aeroallergen sensitization, atopic dermatitis symptoms in the previous 12 months, and EASI score

| Urinary Paraben | Quintile (IQR, ng/mL) | Aeroallergen sensitization | | Atopic dermatitis symptoms in the preceding 12 months | | EASI score | |
| --- | --- | --- | --- | --- | --- | --- | --- |
|  |  | aOR (95% CI) | *P* value | aOR (95% CI) | *P* value | aOR (95% CI) | *P* value |
| Methyl-  paraben | Q1  (0.000-0.479) | Ref. |  | Ref. |  | Ref. |  |
|  | Q2  (2.908-6.602) | 0.853 (0.459-1.585) | 0.615 | 0.785 (0.412-1.498) | 0.463 | 1.022 (0.447-2.339) | 0.958 |
|  | Q3  (10.827-16.809) | 0.657 (0.354-1.218) | 0.182 | 0.701 (0.363-1.355) | 0.291 | 1.110 (0.483-2.553) | 0.805 |
|  | Q4  (28.771-59.904) | 0.918 (0.490-1.718) | 0.789 | 0.829 (0.433-1.587) | 0.571 | 1.346 (0.602-3.012) | 0.469 |
|  | Q5  (193.313-615.517) | 0.547 (0.293-1.021) | 0.058 | 0.642 (0.328-1.259) | 0.197 | 0.670 (0.272-1.652) | 0.385 |
| Ethyl-  paraben | Q1  (0.000-1.273) | Ref. |  | Ref. |  | Ref. |  |
|  | Q2  (2.877-5.318) | 1.045 (0.570-1.915) | 0.887 | 1.417 (0.725-2.768) | 0.308 | 1.450 (0.597-3.526) | 0.412 |
|  | Q3  (11.082-25.060) | 1.212 (0.670-2.191) | 0.525 | 1.010 (0.514-1.984) | 0.977 | 1.125 (0.458-2.763) | 0.797 |
|  | Q4  (46.8202-94.205) | 1.058 (0.580-1.930) | 0.855 | 1.274 (0.653-2.484) | 0.477 | 2.019 (0.868-4.699) | 0.103 |
|  | Q5  (165.087-498.889) | 1.450 (0.787-2.671) | 0.234 | 0.535 (0.633-2.411) | 0.535 | 1.552 (0.651-3.700) | 0.321 |
| Butyl-paraben | Q1  (0.000-0.000) | Ref. |  | Ref. |  | Ref. |  |
|  | Q2  (0.126-0.359) | 1.020 (0.473-2.197) | 0.960 | 1.646 (0.757-3.580) | 0.208 | 1.040 (0.475-2.274) | 0.922 |
|  | Q3  (0.666-1.006) | 0.652 (0.279-1.523) | 0.324 | 1.452 (0.752-2.803) | 0.266 | 0.758 (0.338-1.699) | 0.501 |
|  | Q4  (1.450-2.212) | 1.327 (0.628-2.806) | 0.459 | 1.147 (0.584-2.254) | 0.691 | 1.006 (0.466-2.171) | 0.987 |
|  | Q5  (3.450-6.030) | 0.501 (0.203-1.236) | 0.133 | 0.966 (0.482-1.937) | 0.922 | 0.653 (0.279-1.526) | 0.325 |

CI, confidence interval; EASI, Eczema Area and Severity Index; IQR, interquartile range; Ref., reference group; Q1, first quintile; Q2, second quintile; Q3, third quintile; Q4, fourth quintile; Q5, fifth quintile.

Limit of quantification (LOQ) of all tested parabens is 0.1 μg/L.

*Logistic regression analysis was performed with adjustment for confounding factors, including age, gender, body mass index z-score, the presence of visible mold at home, and exposure to environmental tobacco smoke.

**Supplementary Table 3.** List of significantly different urinary metabolites between the lowest and highest quintile groups of urinary propyl-paraben.

| Metabolism | | Name | Fold change^*^ | *P* value | FDR-adjusted  *P* value | MSI level^†^ |
| --- | --- | --- | --- | --- | --- | --- |
| Amino acid metabolism | Aspartate metabolism | Asparagine | 0.75 | < 0.001 | < 0.001 | 2 |
|  | Glycine and serine metabolism | Threonine | 1.21 | 0.032 | 0.114 | 2 |
|  |  | Serine | 1.26 | < 0.001 | < 0.001 | 1 |
|  |  | Glycine | 1.14 | < 0.001 | 0.018 | 1 |
|  | Urea cycle | Ornithine | 1.62 | 0.039 | 0.114 | 2 |
|  | Branched-chain amino acid metabolism | Isoleucine | 5.55 | < 0.001 | < 0.001 | 2 |
|  | AhR signaling pathway | Picolinic acid | 1.69 | < 0.001 | 0.001 | 1 |
| Lipid metabolism | PPAR signaling pathway | Palmitic Acid | 0.78 | < 0.001 | < 0.001 | 1 |
|  | Fatty acid metabolism | 2-Palmitoylglycerol | 0.80 | 0.001 | < 0.001 | 2 |
| Carbohydrate metabolism | Monosaccharide metabolism | Arabinofuranose | 1.23 | 0.031 | 0.037 | 2 |
|  |  | D-lyxofuranose | 1.93 | < 0.001 | < 0.001 | 2 |
|  |  | Ethyl d-galactofuranoside | 1.11 | 0.023 | 0.058 | 2 |
|  |  | Ribose | 1.13 | 0.021 | 0.058 | 2 |
|  | Glycolysis | 2,3-Dihydroxybutanoic acid | 1.15 | 0.029 | 0.067 | 2 |
| Energy metabolism | TCA cycle | Citrate | 1.23 | < 0.001 | 0.008 | 2 |

AhR, aryl hydrocarbon receptor; PPAR, peroxisome proliferator-activated receptors; TCA, tricarboxylic acid; FDR, false discovery rate; MSI, Metabolomics Standards Initiative.

^*^Fold change was calculated from the mean peak area values of the highest and lowest quintile groups of urinary propyl-paraben.

^†^Definition of MSI levels were defined as level 1 in identified metabolites and level 2 in putatively annotated compounds.

**Supplementary Figure 1** Distribution of urinary propyl-paraben concentrations classified according to the quintile levels.


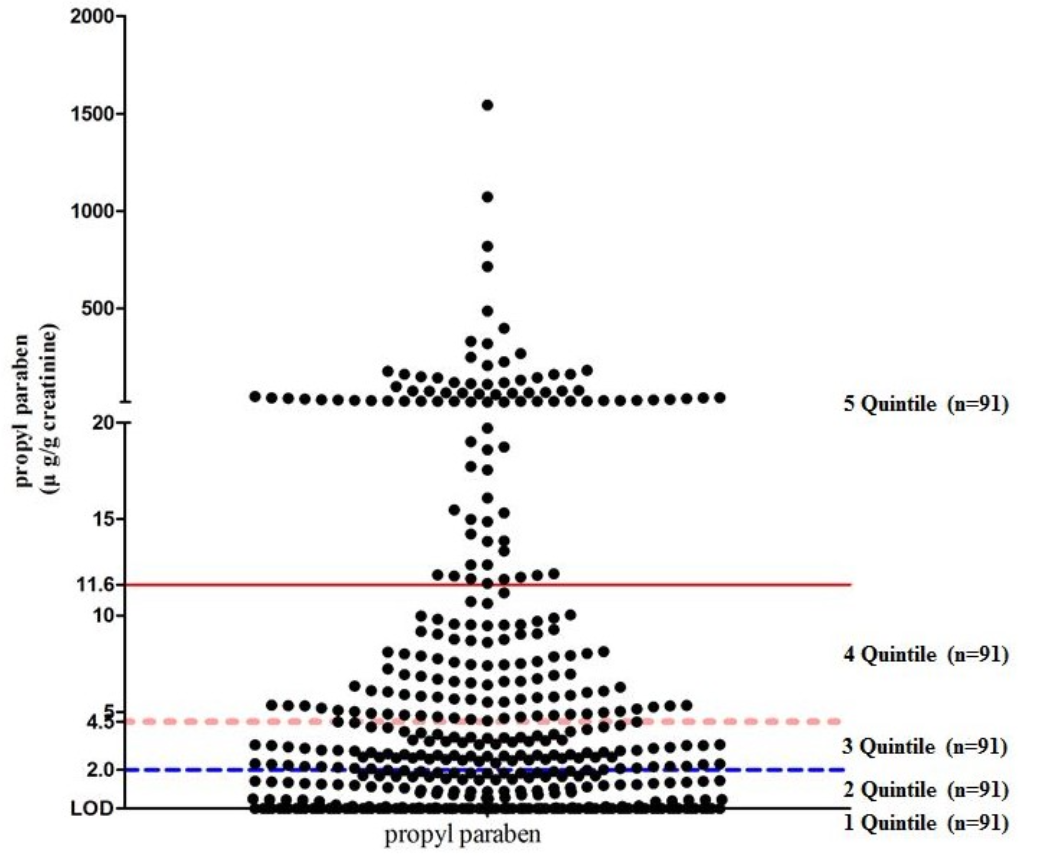


*Each line represents median values in each quintile group of urinary propyl-paraben.

LOD, limit of detection

The figure was processed using Graph 9.0 (https://www.graphpad.com/scientific-software/prism).

**Supplementary Figure 2** Univariate and multivariate analysis of the untargeted metabolomics. (**A)** PCA plot based on the data between the highest and lowest quintile groups of urinary propyl-paraben level. (**B)** Volcano plot of metabolite features. The data were converted to a logarithmic scale, the x-axis shows fold change, and the y-axis is the *P* value. Red circles indicate metabolites that have changed significantly between the two groups. The name of metabolites with *P* value of < 0.001 are represented on the volcano plot.

**
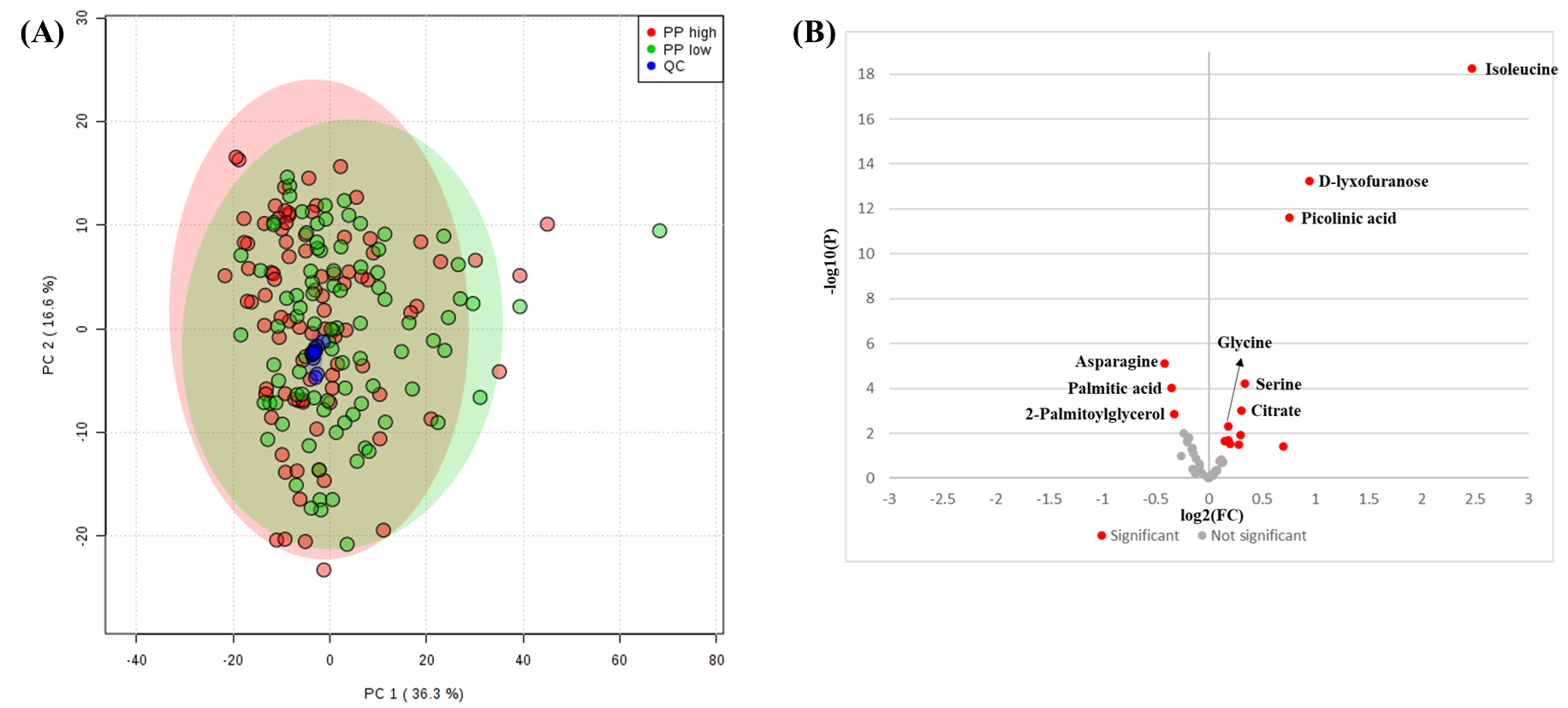
**

The PCA plot was processed using Metaboanalyst 4.0 (https://www.graphpad.com/scientific-software/prism).

QC, quality control; FC, fold change; PP, propyl paraben; PC, principle component.

**Supplementary Figure 3.** Metabolite sets enrichment analysis for the significant urinary metabolites. The width of each bar in the graph indicates the size of the fold enrichment. The colors on the bar graph represent the *P* value of each metabolic pathway.

*
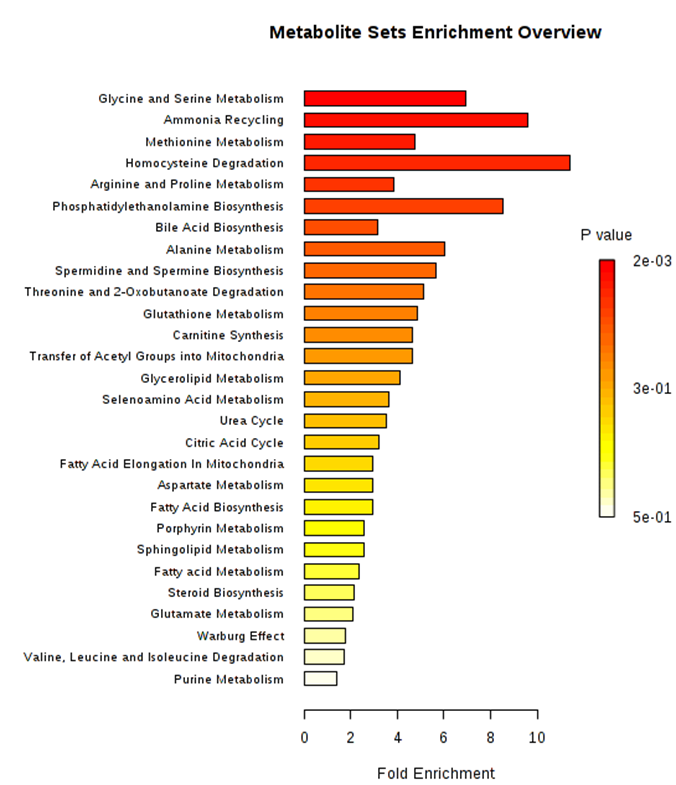
*

**Supplementary Figure 4** Random forest on the degree of difference in the metabolites that were found to be significantly different between the highest and lowest quintile groups of urinary propyl-paraben


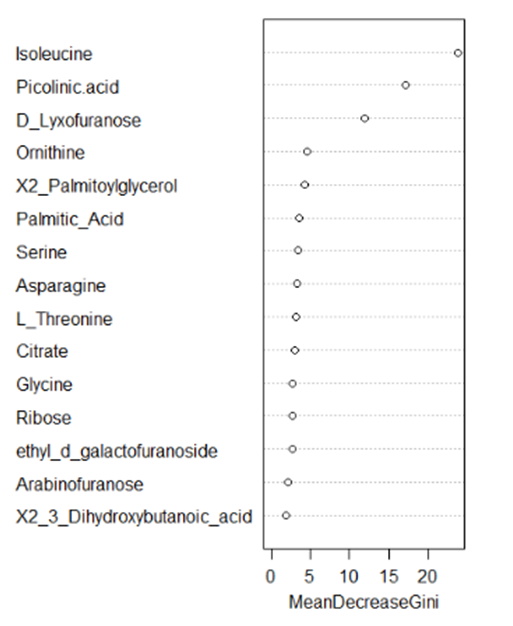


**Supplementary Figure 5** Sumamry of the possible pathways underlying the associations of urinary propyl-paraben with increased EASI score


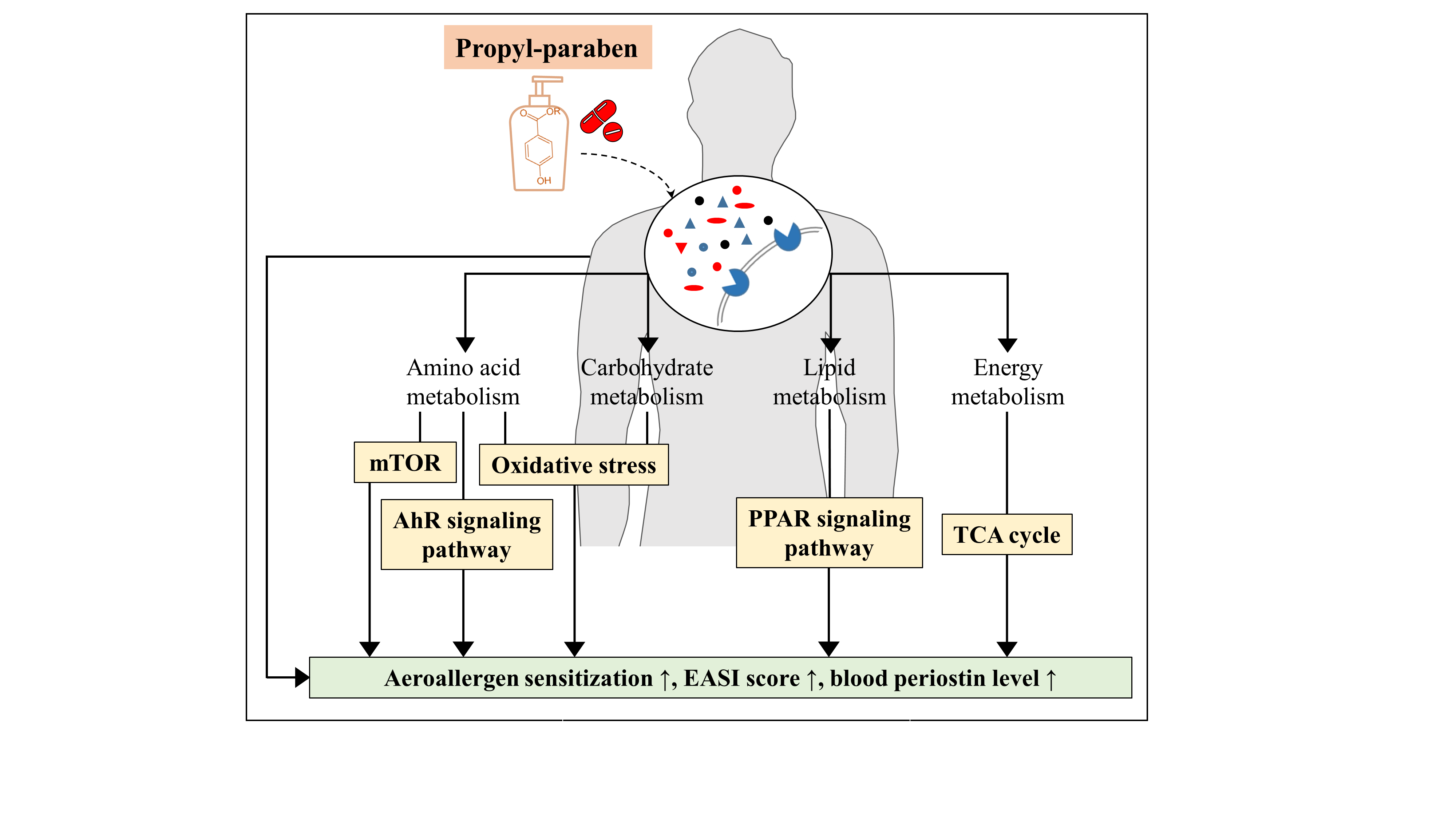

Supplement: Supplementary file 1 — Supplementary Information. [file 41598_2021_83288_MOESM1_ESM.doc]
